# Supplementary material for: Polyphenolic Content and Antimicrobial Effects of Plant Extracts as Adjuncts for Craft Herbal Beer Stabilization
Source: Foods. 2024 Sep 3;13(17):2804. doi: 10.3390/foods13172804 (PMC11395130; doi:10.3390/foods13172804)
Supplement: Supplementary file 1 [file foods-13-02804-s001.zip › foods-3168973-supplementary.pdf]

A

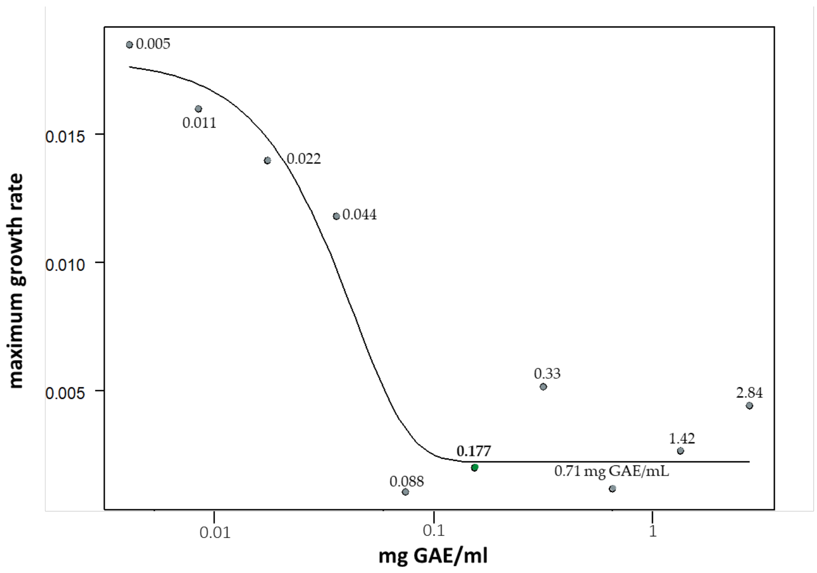

maximum growth rate
0.015
0.010
0.005
0.005
0.011
0.022
0.044
0.33
2.84
1.42
0.177
0.71 mg GAE/mL
0.088
0.01
0.1
1
mg GAE/ml

B

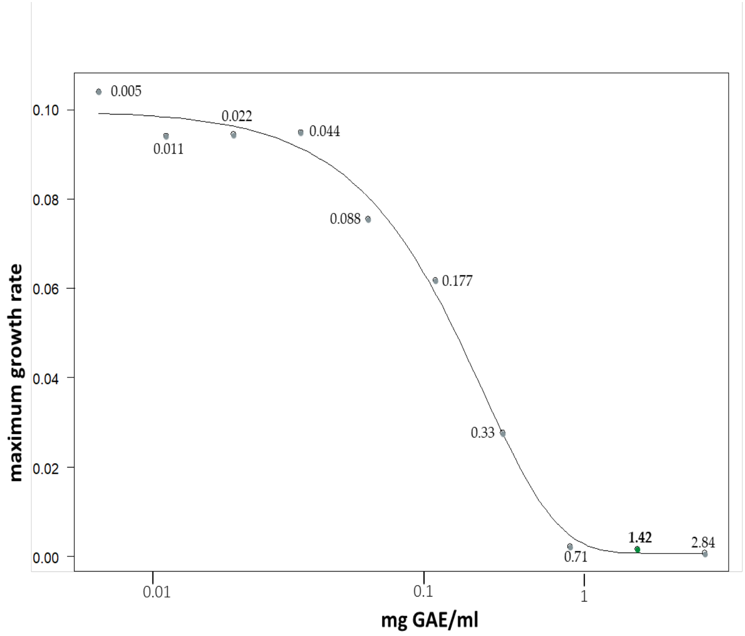

maximum growth rate
0.10
0.08
0.06
0.04
0.02
0.00
0.005
0.011
0.022
0.044
0.088
0.177
0.33
0.71
1.42
2.84
0.01
0.1
1
mg GAE/ml

C

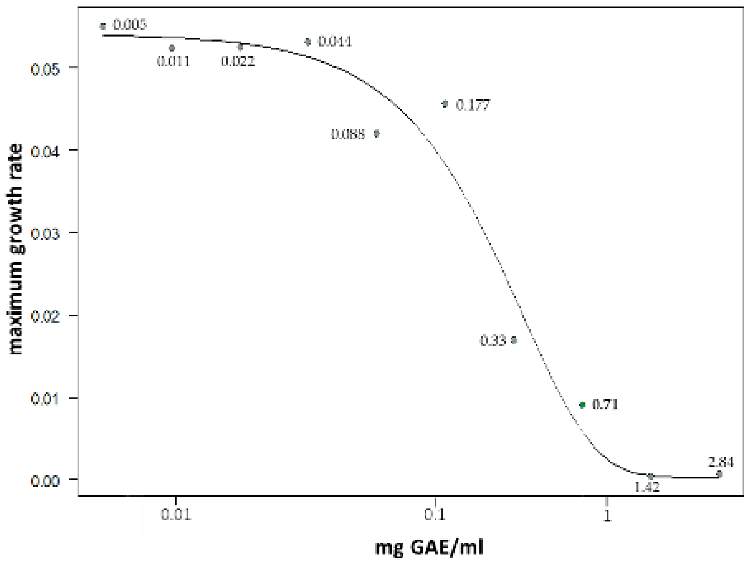

maximum growth rate
0.05
0.04
0.03
0.02
0.01
0.00
0.005
0.011
0.022
0.044
0.177
0.088
0.33
0.71
2.84
1.42
0.01
0.1
1
mg GAE/ml

Figure S1. The effect of polyphenol concentration on the maximum growth rate of spoilage and pathogenic microorganisms. The growth of *S. aureus* DSM20231 was found to be inhibited by the *M. communis* extract at the predicted concentrations of 0.177 ± 0.071 mg GAE/mL (A). *L. casei* ATCC393 growth was inhibited at 1.42 ± 0.81 mg GAE/mL (B), and *L. plantarum* ATCC8014 at 0.71 ± 0.23mg GAE/mL (C).

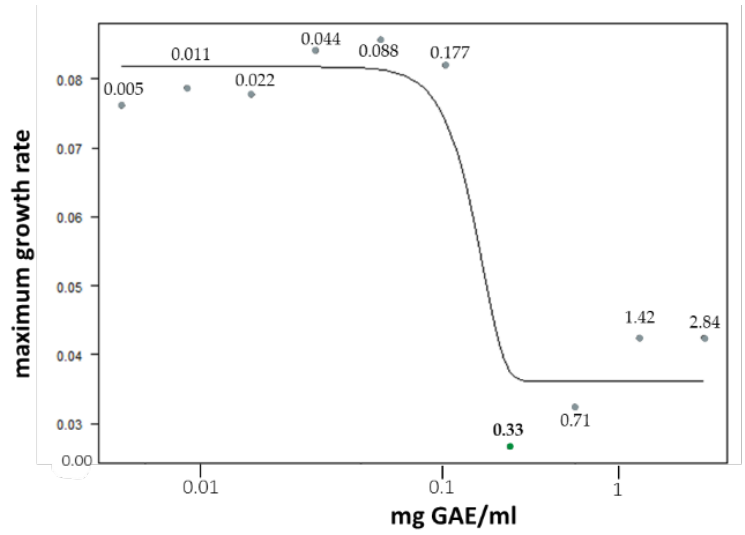

(A)

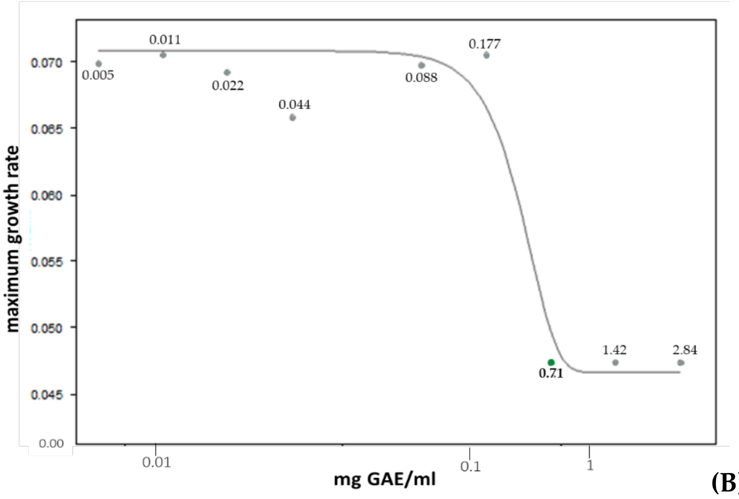

(B)

Figure S2. The effect of polyphenol concentration on the maximum growth rate. *L. brevis* DSM6235 (A) and *F. lindneri* DSM20692 (B) was not completely inhibited by any of the tested concentrations of *M. communis* extract, even though a significant reduction of their specific growth rate (>0.01) was predicted at 0.335 ± 0.85 mg GAE/mL (A) and 0.71 ± 0,25 mg GAE/mL (B), respectively.

Table S1. LCQTOF Mass chromatographic characteristics of compounds tentatively annotated in the Myrtus communis extract.

Compounds	Raw Formula	Maasured mass (*m*/*z*) and adduct	Retention time (min)	Area	Area % (n=3)
Quinic acid Isomer 1	$C_7H_{12}O_6$	193,0709 $(M+H)^+$	1.45	431199343	1,666±0.091
Quinic acid Isomer 2	$C_7H_{12}O_6$	193,0713 $(M+H)^+$	1.663	11588339	0,209±0.015
Galloyl quinic acid	$C_{14}H_{16}O_{10}$	367,0645 $(M+Na)^+$	2.135	1450569	3,578±0.478
Myricetin galloyl hexoside	$C_{28}H_{24}O_{17}$	655.0915 $(M+Na)^+$	2.347	24885343	7,082±0.971
Myricetin galloyl rhamnopyranoside	$C_{28}H_{24}O_{16}$	617,1129 $(M+H)^+$	2.607	49252934	4,227±0.713
Digalloyl-hexahydroxydiphenoyl-glucose	$C_{41}H_{28}O_{27}$	953,0898 $(M+H)^+$	3.08	29395900	2,827±0.125
Unknown			3.623	19659809	11,214±1.655
Myricetin galactoside	$C_{21}H_{20}O_{13}$	481.0979 $(M+H)^+$	5.052	77987409	3,030±0.489
Unknown			9.067	21072297	6,065±0.687
Gallomyrtucommulone C	$C_{27}H_{36}O_{13}$	569,2235 $(M+H)^+$	9.693	42178233	20,302±2.223
Unknown			10.318	141189741	6,189±0.713
Gallomyrtucommulone A	$C_{27}H_{38}O_{13}$	571,2390 $(M+H)^+$	10.661	43041079	2,007±0.905
Unknown			11.24	13954538	2,963±0.348
Unknown			11.44	20605377	2,411±0.221
Unknown			11.854	16764250	2,890±0.845
Unknown			12.987	20097332	3,114±0.458
Unknown			13.932	21656860	4,706±0.378
Unknown			15.042	32728870	4,153±0.698
Unknown			15.904	28879546	4,152±0.359
Vitexin	$C_{21}H_{20}O_{10}$	433,1135 $(M+H)^+$	16.648	34929655	3,387±0.825
Unknown			17.002	23554097	3,253±1.085
Unknown			18.809	22619885	4,730±0.688
Unknown			19.045	32892977	1,666±0.457
